# Associations of Perfluoroalkyl Substances (PFAS) with Lower Birth Weight: An Evaluation of Potential Confounding by Glomerular Filtration Rate Using a Physiologically Based Pharmacokinetic Model (PBPK)

**DOI:** 10.1289/ehp.1408837

**Published:** 2015-05-22

**Authors:** Marc-André Verner, Anne E. Loccisano, Nils-Halvdan Morken, Miyoung Yoon, Huali Wu, Robin McDougall, Mildred Maisonet, Michele Marcus, Reiko Kishi, Chihiro Miyashita, Mei-Huei Chen, Wu-Shiun Hsieh, Melvin E. Andersen, Harvey J. Clewell, Matthew P. Longnecker

**Affiliations:** 1 Department of Environmental Health, Harvard T.H. Chan School of Public Health, Boston, Massachusetts, USA; 2 Institute of Environmental Medicine, Karolinska Institutet, Stockholm, Sweden; 3 Center for Human Health Assessment, The Hamner Institutes for Health Sciences, Research Triangle Park, North Carolina, USA; 4 Department of Global Public Health and Primary Care, University of Bergen, Bergen, Norway; 5 Department of Obstetrics and Gynecology, Haukeland University Hospital, Bergen, Norway; 6 Aegis Technologies, Huntsville, Alabama, USA; 7 Biostatistics and Epidemiology Department, College of Public Health, East Tennessee State University, Johnson City, Tennessee, USA; 8 Department of Epidemiology, Rollins School of Public Health, Emory University, Atlanta, Georgia, USA; 9 Center for Environmental and Health Sciences, Hokkaido University, Sapporo, Japan; 10 Department of Pediatrics, National Taiwan University Hospital, National Taiwan University College of Medicine, Taipei, Taiwan; 11 Epidemiology Branch, National Institute of Environmental Health Sciences, National Institutes of Health, Department of Health and Human Services, Research Triangle Park, North Carolina, USA

## Abstract

**Background:**

Prenatal exposure to perfluoroalkyl substances (PFAS) has been associated with lower birth weight in epidemiologic studies. This association could be attributable to glomerular filtration rate (GFR), which is related to PFAS concentration and birth weight.

**Objectives:**

We used a physiologically based pharmacokinetic (PBPK) model of pregnancy to assess how much of the PFAS–birth weight association observed in epidemiologic studies might be attributable to GFR.

**Methods:**

We modified a PBPK model to reflect the association of GFR with birth weight (estimated from three studies of GFR and birth weight) and used it to simulate PFAS concentrations in maternal and cord plasma. The model was run 250,000 times, with variation in parameters, to simulate a population. Simulated data were analyzed to evaluate the association between PFAS levels and birth weight due to GFR. We compared simulated estimates with those from a meta-analysis of epidemiologic data.

**Results:**

The reduction in birth weight for each 1-ng/mL increase in simulated cord plasma for perfluorooctane sulfonate (PFOS) was 2.72 g (95% CI: –3.40, –2.04), and for perfluorooctanoic acid (PFOA) was 7.13 g (95% CI: –8.46, –5.80); results based on maternal plasma at term were similar. Results were sensitive to variations in PFAS level distributions and the strength of the GFR–birth weight association. In comparison, our meta-analysis of epidemiologic studies suggested that each 1-ng/mL increase in prenatal PFOS and PFOA levels was associated with 5.00 g (95% CI: –21.66, –7.78) and 14.72 g (95% CI: –8.92, –1.09) reductions in birth weight, respectively.

**Conclusion:**

Results of our simulations suggest that a substantial proportion of the association between prenatal PFAS and birth weight may be attributable to confounding by GFR and that confounding by GFR may be more important in studies with sample collection later in pregnancy.

**Citation:**

Verner MA, Loccisano AE, Morken NH, Yoon M, Wu H, McDougall R, Maisonet M, Marcus M, Kishi R, Miyashita C, Chen MH, Hsieh WS, Andersen ME, Clewell HJ III, Longnecker MP. 2015. Associations of perfluoroalkyl substances (PFAS) with lower birth weight: an evaluation of potential confounding by glomerular filtration rate using a physiologically based pharmacokinetic model (PBPK). Environ Health Perspect 123:1317–1324; http://dx.doi.org/10.1289/ehp.1408837

## Introduction

Perfluoroalkyl substances (PFAS) are synthetic compounds that are resistant to degradation and have been found worldwide in environmental media and biota, including humans. The most widely studied PFAS are perfluorooctane sulfonate (PFOS) and perfluorooctanoic acid (PFOA). PFOS was an ingredient in the Scotchgard stain repellent manufactured by 3M, but the company decided to stop producing PFOS in 2002 after it had been found in wildlife and humans (Agency for Toxic Substances and Disease Registry 2009). PFOA is a surfactant that is used in the production of many consumer goods, including nonstick coating in cookware. The eight major companies producing or using PFOA have agreed to work toward eliminating emissions and product content of PFOA by 2015 [U.S. Environmental Protection Agency (EPA) 2006]. Despite the reductions in the production and emission of PFOS and PFOA, these persistent compounds can still be detected in biological samples from the general population. For example, PFOS and PFOA have been detected in the blood of > 98% of participants in the 2009–2010 U.S. National Health and Nutrition Examination Survey (NHANES) [Centers for Disease Control and Prevention (CDC) 2013] and 2009–2011 Canadian Health Measure Survey (CHMS) (Health Canada 2013). PFOS and PFOA have also been detected in maternal blood during pregnancy, cord blood at delivery, and breast milk (Kim SK et al. 2011; Olsen et al. 2009), indicating that humans are exposed during critical prenatal and early postnatal windows of development.

Many epidemiologic studies have reported an association between maternal and cord blood PFAS levels and reductions in birth weight (Apelberg et al. 2007; Chen et al. 2012; Fei et al. 2007; Maisonet et al. 2012; Washino et al. 2009; Whitworth et al. 2012). Although these studies accounted for potential confounding by many variables, none adjusted for glomerular filtration rate (GFR). GFR, the flow rate of fluid being filtrated by the kidneys, increases by about 50% during the first half of pregnancy and declines slightly during the second half of pregnancy (Gibson 1973). Two studies of GFR during pregnancy have shown that women whose GFR fails to rise sufficiently during pregnancy tend to have smaller babies (Gibson 1973; Morken et al. 2014). On the other hand, GFR is likely to influence the urinary excretion of xenobiotics like PFAS. Indeed, higher blood PFAS levels have been observed in people with lower GFR (Shankar et al. 2011; Watkins et al. 2013). Watkins et al. (2013) evaluated the direction of the association between PFOA and reduced kidney function (indicated by GFR) by comparing results obtained with measured serum PFOA levels (which could be influenced by GFR) and estimated serum PFOA levels (which were independent of GFR): An association was observed only with measured PFOA, suggesting that the association may be a consequence of, rather than a cause of, decreased kidney function. If so, women with lower GFR during pregnancy would tend to have smaller babies and higher blood PFAS levels. This raises the possibility that GFR confounds the association between prenatal PFAS exposure and birth weight. To what extent GFR influences this association has yet to be evaluated.

In this study, we assessed how much of the epidemiologic association between prenatal PFOS and PFOA (PFAS thereafter) exposure and birth weight could be attributable to confounding by GFR. We modified a recently developed physiologically based pharmacokinetic (PBPK) model of PFAS during pregnancy (Loccisano et al. 2013) to reflect the association between GFR and PFAS levels and birth weight. The model was run repeatedly, using Monte Carlo simulation techniques, with variation in parameters, to simulate a population. Estimates of the birth weight–PFAS association obtained from simulated PFAS levels and birth weight were subsequently compared with estimates from a meta-analysis of existing epidemiologic studies.

## Methods

*Overview.* We used a PBPK model to run Monte Carlo simulations of a study population and to generate pairs of predictions for PFAS level and birth weight. PBPK-derived estimates were subsequently analyzed by linear regression. We also performed a meta-analysis of published epidemiologic studies of prenatal PFAS exposure and birth weight to obtain summary effect estimates. Results obtained from simulated PFAS levels and birth weights were compared with results from our meta-analysis to evaluate how much of this association might be attributable to the influence of GFR.

**Figure 1:**
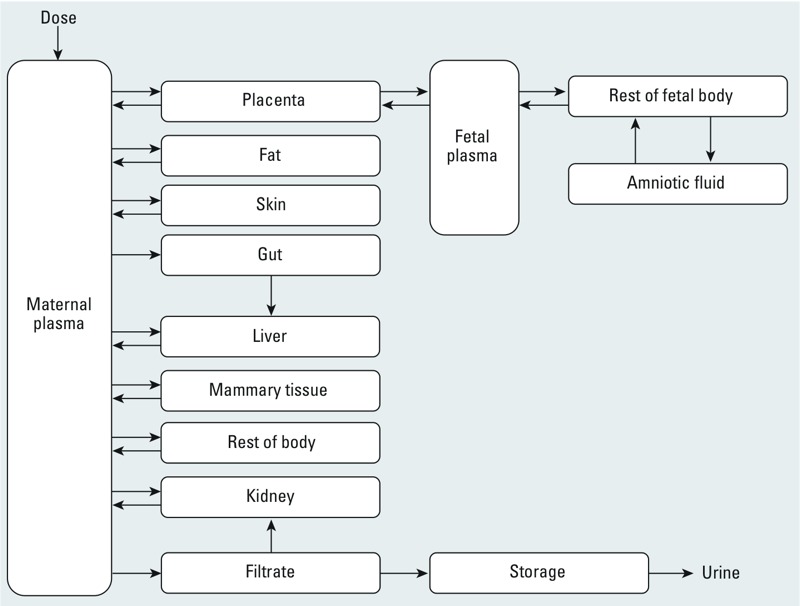
Structure of human gestation PBPK model for PFOS and PFOA adapted from Loccisano et al. (2013) with permission of Taylor & Francis LLC.

*The PBPK model.* We modified a published PBPK model of PFOA and PFOS during pregnancy (Loccisano et al. 2013). This multi-compartment model included maternal compartments (plasma, liver, fat, gut, skin, mammary, rest of body, kidney, filtrate, and storage) and the placenta, fetal plasma, rest of fetal body, and amniotic fluid (Figure 1). Exposure to PFAS was modeled as an input into the maternal plasma compartment to encompass absorbed doses through different routes. Distribution in the different compartments was driven by blood flow rates in and out of compartments, tissue volume, and tissue:blood partition coefficients. PFAS excretion in urine was modeled as a multi-step process: The free (unbound) PFAS in plasma was first filtered through the kidneys followed by extensive active reabsorption, with the unreabsorbed fraction continuing its way to a storage compartment before excretion. We updated the description of placental blood flow and fetal cardiac output according to equations presented by Yoon et al. (2011). The modified version of the PBPK model code is provided in Supplemental Material, “PBPK Model Code.” A conceptual representation with basic mass-balance differential equations is also provided in Supplemental Material, Figure S1.

We also modified the model so that the initial body burden (at the beginning of pregnancy) and intake rate during pregnancy are calculated based on an initial plasma PFAS level [C_initial_ (nanograms per milliliter)]. The initial amount of PFAS in the different maternal tissues (Amount_t_) at each Monte Carlo simulation (i) was computed as the product of the initial plasma PFAS level (C_initial_), the tissue:plasma partition coefficient (Partition_t_) and the tissue volume (Volume_t_):

Amount_t(i)_ = C_initial(i)_ × Partition_t(i)_ × Volume_t(i)_. [1]

Maternal PFAS intake rate during pregnancy was estimated from initial plasma PFAS level. To estimate maternal PFAS intake rate during pregnancy, we assumed the initial plasma PFAS level to be at steady state. The hourly intake rate was calculated accordingly using a rearrangement of a classic steady state equation that accounts for compound-specific half-life (hours), volume of distribution (liters), and dosing interval (hours) (Dhillon and Kostrzewski 2006):

Intake (ng/hr)_(i)_ = C_initial(i)_ × Volume of distribution_(i)_ × Dosing interval × ln(2)/Half-life, [2]

where the volume of distribution was calculated based on partition coefficients and organ volumes, the dosing interval was 1 hr (simulation time increment), and the half-lives of PFOS and PFOA were 47,304 hr (5.4 years) and 33,288 hr (3.8 years) (Olsen et al. 2007).

To parameterize the relationship between GFR and birth weight, we performed a meta-analysis of three studies where individual-specific paired GFR and birth weight measurements were available in the publication or made available to us (Dunlop 1981; Gibson 1973; Morken et al. 2014). Other studies of GFR or indicators of GFR (e.g., serum creatinine, serum uric acid) and birth weight were identified but did not report individual-specific data or regression coefficients and, consequently, could not be used in our meta-analysis (Akahori et al. 2012; Davison and Hytten 1974; Dunlop et al. 1978; Duvekot et al. 1995; Knopp et al. 1985; Laughon et al. 2009). Because GFR changes during pregnancy and the measurements were taken at different times during pregnancy, we calculated standardized GFR values (GFR_ratio_) as the ratio of the observed GFR for each subject to the mean GFR at that gestational age [Gibson 1973 (28 gestational weeks); Dunlop 1981 (26 gestational weeks); Morken et al. 2014 (mean, 18 gestational weeks)]. We computed the coefficient relating birth weight to GFR_ratio_ as the inverse variance–weighted average of the coefficient based on regression models of data from Gibson (1973) (*n* = 20), Dunlop (1981) (*n* = 25), and Morken et al. (2014) (*n* = 953). The raw data from these studies were either presented in the original publication (Gibson 1973; Dunlop 1981) or were available to us (Morken et al. 2014). In the first two studies, GFR was measured using inulin clearance. In the third study, GFR was estimated based on plasma creatinine and the Cockroft–Gault formula (Koetje et al. 2011). A separate multiple regression model of birth weight was fitted for each study; all models were adjusted for gestational age at birth. The Morken et al. (2014) data were additionally adjusted for prepregnancy body weight and sampling strata. Because estimation of GFR on the basis of a single measure of plasma creatinine is known to be imprecise (Aras et al. 2012), the coefficient for GFR_ratio_ from the Morken et al. (2014) study was deattenuated to account for the effect of measurement error (Willett 1990), by dividing by an intraclass correlation coefficient of 0.76 for serum creatinine (Al-Delaimy et al. 2006) before calculating the overall inverse-variance weighted average. Each unit increase in GFR_ratio_ was associated with an increase in birth weight (± SE) of 67 ± 535 g in the Dunlop (1981) study, 1,603 ± 784 g in the Gibson (1973) study, and 164 ± 77 g in the Morken et al. (2014) study. The meta-analytic coefficient was a 175.5 ± 75.9-g increase in birth weight per unit increase in GFR_ratio_.

We used a two-tier approach to generate variability in GFR_ratio_ and induce an association between GFR_ratio_ and birth weight in Monte Carlo simulations. For each Monte Carlo simulation (i), we first sampled a GFR_ratio_ value from the distribution of GFR_ratio_ in the data of Morken et al. (2014) [mean ± SD, 1.0 ± 0.246; range, 0.508–1.492 (± 2 SDs)]. The SD from Morken et al. (2014) was selected because in this more recent study, the distribution of GFR_ratio_ was considered to be more relevant because of the increase in prevalence of overweight and obesity and the correlation of GFR with body mass index (Bosma et al. 2004). During each simulation, the time-course of GFR (GFR_t_) during pregnancy was obtained by multiplying the reference gestational GFR_t_ profile (GFR as a function of time elapsed since conception, as described in the original PBPK model) by the sampled GFR_ratio_:

GFR_t(i)_ = GFR_ratio(i)_ × Reference gestational GFR_t_. [3]

Then we calculated a birth weight according to the meta-analytic regression between GFR_ratio_ and birth weight derived from three studies, as described above. This was accomplished by using the equation derived from the aforementioned regression and randomly sampling an error term based on the distribution of residuals:

Calculated birth weight (g)_(i)_ = Intercept + β × GFR_ratio(i)_ + Residual_(i)_, [4]

where the intercept was 3,376 g, the β was 175.5 g per 1-unit increase in GFR_ratio_, and the residual was sampled from a distribution with a mean of 0 g, an SD of 441 g, and ranging from –882 g to 882 g (± 2 SDs). Fetal growth in the original PBPK model was described using a time-dependent fetal growth curve (Loccisano et al. 2013). We adjusted this standard fetal growth curve to match the calculated birth weight from Equation 4. To do so, we multiplied the standard fetal growth curve (reference fetal weight_t_) by the ratio of calculated birth weight on the reference fetal weight_t_ at delivery (3,509 g). For each simulation (i), the time-course of fetal weight (fetal weight_t_) was described using the following equation:

Fetal weight_t(i)_ = (Calculated birth weight_(i)_/3,509 g) × Reference fetal weight_t_. [5]

*PBPK model global sensitivity analysis.* Because the PBPK model used herein incorporates > 40 parameters that can vary within a population (e.g., volume of organs, perfusion rates, tissue:plasma partition coefficients), we first ran a sensitivity analysis to identify parameters with the highest relative influence on maternal plasma PFAS levels across pregnancy and cord plasma PFAS levels at delivery. We opted for the Morris global method, which evaluates parameter sensitivity over a range of physiological scenarios by taking the mean of many local sensitivity analyses calculated over the entire parameter space, thus accounting for interactions (McNally et al. 2011). We allowed parameters to vary between 70% and 130% of their mean value—a 15% coefficient of variation with bounds at ± 2 SDs. For this exercise, we used initial maternal plasma levels of 13.02 ng/mL for PFOS and 2.53 ng/mL for PFOA to reflect levels in published epidemiologic studies as noted below in the “Monte Carlo simulations” section. Sensitivity coefficients were calculated by adapting the M code of the Morris Test included in the acslX Optimum suite of tools (Aegis Technologies Inc., Huntsville, AL, USA) to our study. The set of most influential parameters—those for which small perturbations have the most significant effect on PFOS and PFOA levels (coefficient within a factor of 10 of the most sensitive model parameter at any month of pregnancy or at delivery)—were allowed to vary in the Monte Carlo analyses.

*Assessment of PBPK model accuracy.* To assess how well the model describes the pharmacokinetics of PFAS during pregnancy, we compared simulated plasma PFAS profiles with observed serial levels. We identified two reports with data that were not used by Loccisano et al. (2013) for model development and met the following criteria: presented two serial maternal blood PFAS levels, and presented sufficient information on sample collection times (Glynn et al. 2012; Monroy et al. 2008). For each of the two reports and each PFAS (PFOS and PFOA), we performed 10,000 Monte Carlo iterations. At each Monte Carlo iteration, the model *a*) sampled values for sensitive parameters identified in the global sensitivity (Table 1); *b*) sampled a plasma PFAS level from the published distributions at the first blood sample collection time point; *c*) adjusted the initial plasma level (at the time of conception), by iterative model simulations, to obtain matching simulated and sampled PFAS level at the time of the first blood sample collection (tolerance: 0.1%); and *d*) simulated a complete pharmacokinetic profile based on the initial plasma level. We visually compared the distribution of simulated plasma PFAS profiles from the Monte Carlo iterations with the distribution of observed PFAS levels in the second blood samples from the two reports mentioned above.

**Table 1:** Distributions of parameters used in the Monte Carlo simulations.

Parameter	PFAS	Mean ± SD	Minimum	Maximum
Standardized glomerular filtration rate (GFR_ratio_)^*a*^	—	1.000 ± 0.246	0.508	1.492
Residual birth weight (g)^*b*^	—	0 ± 441	–882	882
Prepregnancy body weight (kg)^*c*^	—	70.3 ± 14.3	37.0	134.0
Volume of liver as a fraction of body weight^*d*^	—	0.026 ± 0.004	0.018	0.034
Liver:plasma partition coefficient^*d*^	PFOS	3.720 ± 0.558	2.604	4.836
	PFOA	2.200 ± 0.330	1.540	2.860
Rest of body:plasma partition coefficient^*d*^	PFOS	0.200 ± 0.030	0.140	0.260
	PFOA	0.120 ± 0.018	0.084	0.156
Free fraction in maternal plasma^*d*^	PFOS	0.025 ± 0.004	0.017	0.033
	PFOA	0.020 ± 0.003	0.014	0.026
Free fraction in fetal plasma^*d*^	PFOS	0.025 ± 0.004	0.017	0.033
	PFOA	0.020 ± 0.003	0.014	0.026
Resorption maximum velocity (mg/hr/kg^0.75^)^*d*^	PFOS	3.500 ± 0.525	2.450	4.550
	PFOA	10.00 ± 1.50	7.000	13.000
Affinity constant (mg/L)^*d*^	PFOS	0.023 ± 0.003	0.017	0.029
	PFOA	0.055 ± 0.008	0.039	0.071
Initial plasma PFAS levels (ng/mL)	PFOS	13.02 ± 4.79	0.01	100.00
	PFOA	2.53 ± 1.13	0.01	100.00
All distributions were assumed to be normal. Values presented are arithmetic means and SDs. ^***a***^Distribution of GFR_ratio_ pooled from the three selected studies (Dunlop 1981; Gibson 1973; Morken et al. 2014). ^***b***^From the GFR_ratio_–birth weight meta-analytic regression. ^***c***^Distribution of prepregnancy body weight from the Norwegian Mother and Child Cohort Study (MoBa). ^***d***^Mean values taken from Loccisano et al. (2013); SDs were calculated assuming a coefficient of variation of 15%, and bounds were set to ± 2 SD.				

*Monte Carlo simulation.* We used a Monte Carlo procedure to simulate population PFOA and PFOS levels across pregnancy. At each Monte Carlo iteration, the PBPK model sampled values for sensitive parameters identified in the global sensitivity analyses and initial blood PFAS levels from probabilistic distributions (Table 1) before simulation of PFAS levels during the 9 months of pregnancy. To be able to compare results from simulations with those from epidemiologic studies on PFAS and birth weight included in our meta-analysis [described below in “Meta-analysis of PFAS-birth weight epidemiologic studies” (Apelberg et al. 2007; Chen et al. 2012; Fei et al. 2007; Hamm et al. 2010; Maisonet et al. 2012; Washino et al. 2009; Whitworth et al. 2012)], we used initial plasma PFAS distributions based on levels reported in these studies. We calculated the mean PFOS (13.02 ng/mL) and PFOA (2.53 ng/mL) levels by averaging the reported mean or median maternal blood or cord blood levels (studies were weighted equally). These epidemiologic studies reported different measures of spread for blood PFAS levels (i.e., range, standard deviation, geometric standard deviation, interquartile range). Because these measures of spread cannot be directly combined, we derived a standard deviation based on coefficients of variations of 0.37 for PFOS and 0.45 for PFOA calculated using data from Fei et al. (2007), the largest study (*n* = 1,399) included in our meta-analysis (described below). Monthly simulated maternal plasma PFAS levels, simulated cord plasma levels at delivery, and calculated birth weight were collected from simulations to be used in regression models of PFAS and birth weight. We ran 250,000 Monte Carlo iterations to achieve convergence in the PFAS–birth weight linear regression coefficient (β).

*Sensitivity analyses.* We evaluated the influence of different assumptions on the association between PBPK-derived PFAS levels and birth weight. In addition to analyses noted above, we ran multiple Monte Carlo simulations with different parameters for PFAS distributions (higher and lower means and standard deviations) and different coefficients for the GFR–birth weight association. Specifically, we halved or doubled these three parameters, one at a time. We also ran Monte Carlo simulations with different sampling seeds to evaluate reproducibility. We identified two studies that evaluated PFOA half-life in populations exposed through drinking water; Brede et al. (2010) estimated a half-life of 3.26 years, which is similar to the 3.8-year half-life used in our study (Olsen et al. 2007), whereas Bartell et al. (2010) estimated a shorter half-life of 2.3 years. To evaluate the impact of a shorter half-life on our results, additional Monte Carlo simulations were carried out using the half-life reported by Bartell et al. (2010).

*Meta-analysis of PFAS–birth weight epidemiologic studies.* We identified human studies published in English in 2012 or earlier using the PubMed (http://www.ncbi.nlm.nih.gov/pubmed) search terms “birth weight” and “perfluorooctane sulfonate” or “perfluorooctanoic acid.” This identified articles with the search terms in the title, abstract, or key words. To be eligible for inclusion in the analysis, the study had to have results available from a multiple regression model of birth weight (grams) as a function of PFOS or PFOA in nanograms per milliliter concentration in maternal blood from pregnancy or cord blood. In one case (Apelberg et al. 2007), the β coefficient originally published (grams birth weight per interquartile increase in PFAS) was reexpressed as nanograms per milliliter by using the interquartile distance. In three instances we found studies that had fit models similar to what we sought, but the published results could not be reexpressed to obtain a reasonable approximation of what we needed. In these cases we contacted the original authors to obtain the coefficients of interest. Specifically, Washino et al. (2009) and Chen et al. (2012) had fit models with log of PFAS as the independent exposure variable, and Maisonet et al. (2012) had fit the desired model but had not put the β coefficients in the publication. We used these regression coefficients to calculate inverse variance–weighted summary β coefficients for PFOS and PFOA. A list of included and excluded studies and a brief description of each is provided in Supplemental Material, Table S1.

## Results

*PBPK modeling of PFAS levels.* We first performed a Morris global sensitivity analysis to identify sensitive model parameters, where a higher coefficient means greater sensitivity. The following parameters had a sensitivity coefficient within a factor of 10 of the most sensitive parameter at some point during pregnancy or at delivery: prepregnancy body weight, liver volume, liver:plasma partition coefficient, rest of body:plasma partition coefficient, free fraction in maternal and cord plasma, renal reabsorption constant, and maximum reabsorption velocity (sensitivity coefficients are presented in Supplemental Material, Table S2). For example, the most sensitive parameter for PFOS levels in cord plasma was the free fraction in fetal plasma (global sensitivity coefficient = 0.0046). In a one-at-a-time sensitivity analysis, a 10% change in this parameter was associated with an 8.9% change in simulated cord plasma PFOS level. In comparison, a 10% change in the liver volume (global sensitivity coefficient = 0.0003) was associated with a 0.9% change in simulated cord plasma PFOS level.

**Figure 2:**
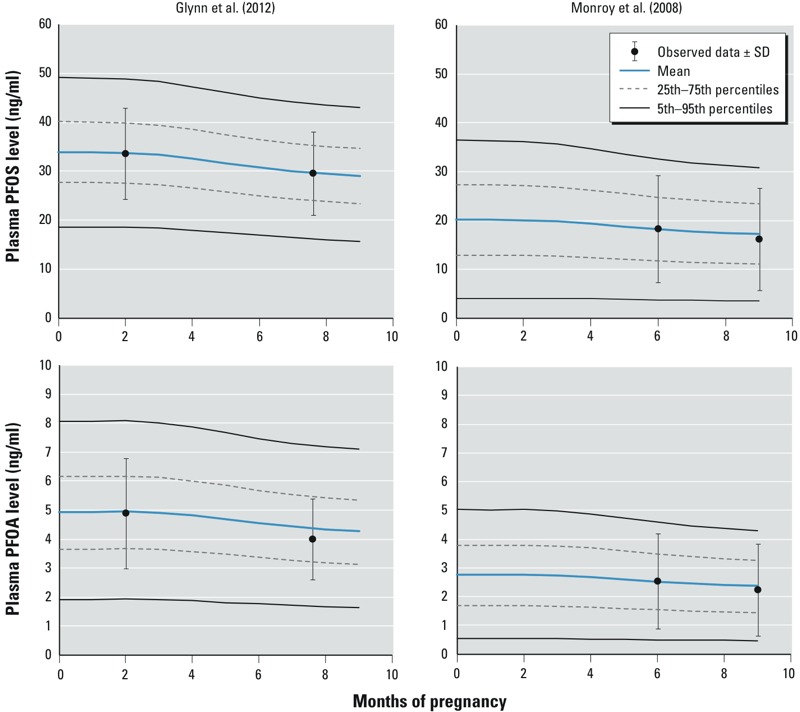
Comparison of simulated versus measured levels from Glynn et al. (2012) and Monroy et al. (2008). Distributions of simulated levels are from 10,000 Monte Carlo simulations.

To assess model accuracy, we simulated maternal plasma PFAS levels based on the first of the two serial measurements of PFAS from two published studies (Glynn et al. 2012; Monroy et al. 2008) and visually compared simulated profiles with observed levels (Figure 2). Simulated and observed PFOS and PFOA levels declined over the course of pregnancy in a similar fashion. However, the model slightly underestimated the decline in PFOA levels from the Glynn et al. (2012) study: Mean simulated PFOA level at the time of second blood draw was 4.3 ng/mL, whereas mean reported level was 4.0 ng/mL.

**Figure 3:**
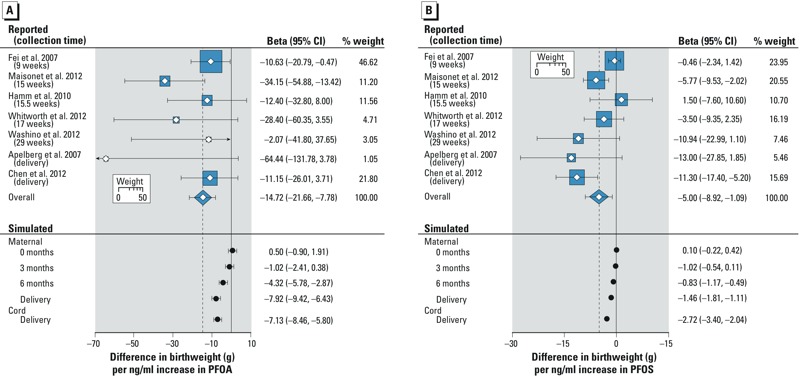
Difference in birth weight (g) per 1-ng/mL increase in reported and simulated PFOA (*A*) and PFOS (*B*) levels. The size of the square represents the weight of each study in the calculation of the overall meta-analytic association. The heterogeneity chi-square for the PFOA meta-analysis was 7.4 (not statistically significant), and for PFOS was 20.1 (*p *< 0.05), both with 6 degrees of freedom. The summary beta coefficient for PFOS was from a random-effects model.

In linear regression analyses, the association between simulated maternal and cord plasma PFAS levels and birth weight was dependent on the time elapsed after conception. For both PFOA (Figure 3A) and PFOS (Figure 3B), the association between simulated maternal plasma levels and birth weight only appeared after the third month of pregnancy and was strongest at the time of delivery. The association between simulated PFOA levels and birth weight was similar for maternal plasma at term [β: –7.9 g; 95% confidence interval (CI): –9.4, –6.4] and cord plasma (β: –7.1 g; 95% CI: –8.5, –5.8). For PFOS, the association between simulated cord plasma levels and birth weight (β: –2.7 g; 95% CI: –3.4, –2.0) was slightly stronger than that estimated based on simulated maternal plasma levels (β: –1.5 g; 95% CI: –1.8, –1.1).

In sensitivity analyses, we evaluated whether the results were robust to changes in initial plasma PFAS level distributions (mean and SD), variations in coefficients for the GFR–birth weight association and different Monte Carlo sampling seeds (reproducibility). These analyses showed that the strength of the simulated PFAS–birth weight association (i.e., confounding by GFR) is influenced by initial plasma PFAS level distributions and the GFR–birth weight coefficient: Stronger associations were obtained with lower mean initial plasma PFAS levels and lower SDs, and with higher GFR–birth weight coefficients (Table 2). When more than one parameter was changed at a time, their influence was additive. As an example, a lower PFOA mean (multiplier = 0.5) and a stronger β for the GFR–birth weight association (multiplier = 2) resulted in a 23.3-g (95% CI: –26.0, –20.6) decrease in birth weight per nanograms per milliliter increase in simulated cord plasma levels; conversely, a higher PFOA mean (multiplier = 2) and a weaker β for the GFR–birth weight association (multiplier = 0.5) resulted in a 2.4-g (95% CI: –3.1, –1.8) decrease in birth weight per nanograms per milliliter increase in simulated cord plasma levels. Results from Monte Carlo simulations using different sampling seeds did not vary substantially, which supports the reproducibility of results (Table 2). Using a shorter half-life of 2.3 years for PFOA (compared with 3.8 in main analyses) increased the strength of the association between simulated levels in maternal plasma at term and birth weight by 21% (β: –9.6 g; 95% CI: –11.0, –8.2) and between simulated levels in cord plasma and birth weight by 14% (β: –8.1 g; 95% CI: –9.4, –6.8).

**Table 2:** Sensitivity analyses evaluating the influence of the PFAS distribution and the strength of the GFR–birth weight association on the simulated change in birth weight (g) per ng/mL increase in PFAS levels attributable to GFR.

Multiplier			Sampling seed	Change in birth weight (g) per ng/mL increase in	
Mean PFAS level^*a*^	Coefficient of variation PFAS levels^*b*^	Beta of the GFR–birth weight association^*c*^		Maternal plasma PFAS level at delivery [β (95% CI)]	Cord plasma PFAS level at delivery [β (95% CI)]
PFOA
1 (main results)	1 (main results)	1 (main results)	123456789	–7.92 (–9.42, –6.43)	–7.13 (–8.46, –5.80)
2	1	1	123456789	–3.96 (–4.70, –3.21)	–3.56 (–4.23, –2.90)
0.5	1	1	123456789	–15.88 (–18.86, –12.89)	–14.28 (–16.95, –11.62)
1	2	1	123456789	–3.29 (–4.19, –2.40)	–3.20 (–4.03, –2.37)
1	0.5	1	123456789	–26.07 (–28.75, –23.39)	–17.59 (–19.67, –15.51)
1	1	2	123456789	–13.40 (–16.80, –14.92)	–11.66 (–13.01, –10.31)
1	1	0.5	123456789	–5.17 (–6.66, –3.68)	–4.86 (–6.18, –3.53)
1	1	1	11111	–8.51 (–10.01, –7.02)	–7.33 (–8.67, –5.99)
1	1	1	99999	–7.77 (–9.27, –6.28)	–6.89 (–8.23, –5.56)
PFOS
1 (main results)	1 (main results)	1 (main results)	123456789	–1.46 (–1.81, –1.11)	–2.72 (–3.40, –2.04)
2	1	1	123456789	–0.73 (–0.91, –0.56)	–1.36 (–1.70, –1.02)
0.5	1	1	123456789	–2.93 (–3.63, –2.23)	–5.45 (–6.81, –4.09)
1	2	1	123456789	–0.54 (–0.75, –0.34)	–1.15 (–1.57, 0.73)
1	0.5	1	123456789	–5.16 (–5.80, –4.51)	–6.60 (–7.65, –5.55)
1	1	2	123456789	–2.77 (–3.12, –2.41)	–5.01 (–5.70, –4.32)
1	1	0.5	123456789	–0.81 (–1.16, –0.46)	–1.57 (–2.25, –0.90)
1	1	1	11111	–1.80 (–2.15, –1.44)	–3.13 (–3.82, –2.45)
1	1	1	99999	–1.42 (–1.77, –1.07)	–2.68 (–3.36, –2.00)
^***a***^Mean values were 2.53 ng/mL for PFOA and 13.02 ng/mL for PFOS in main analyses. ^***b***^Coefficients of variation were 0.446 for PFOA and 0.368 for PFOS in main analyses. ^***c***^The beta in of the GFR–birth weight association was 175.5 g per 1-unit increase GFR_ratio_ in the main analyses.					

*Meta-analysis of epidemiologic studies.* All studies of prenatal PFOA reported an association with reduced birth weight, with β coefficients ranging from –2.1 g to –64.4 g per nanograms per milliliter increase in PFOA levels (Figure 3A). An association between PFOS and reduced birth weight was observed in six of seven studies, with β coefficients ranging from –13.0 g to –0.5 g per nanograms per milliliter increase in PFOS levels (Figure 3B). The summary β coefficients for grams birth weight per nanograms per milliliter increase in PFOA and PFOS levels were –14.7 g (95% CI: –21.7, –7.8) and –5.0 g (95% CI: –8.9, –1.1), respectively.

## Discussion

In this study, we aimed to evaluate how much of the epidemiologic association between prenatal exposure to PFAS and reduced birth weight might be attributable to confounding by GFR. Results from Monte Carlo PBPK model simulations suggest that GFR drives a portion of this association, but not all of it, and that its influence becomes more important with increasing gestational weeks.

When our default assumptions were applied, the association between simulated maternal and cord plasma PFAS levels at the time of delivery and birth weight represented a substantial proportion of the association observed in our meta-analysis of epidemiologic studies. This suggests that epidemiologic studies presented herein, which have not controlled for GFR, might have overestimated the influence of prenatal exposure to PFAS on fetal growth. Our results also suggested that GFR had less influence on PFAS levels in maternal plasma early in pregnancy. In a meta-regression analysis of the epidemiologic data in Figure 3 that we conducted (not shown), week of blood draw was associated with a larger negative coefficient for PFOS (–0.39 g birth weight per nanograms per milliliter increase in PFOS per gestational week, *p* < 0.01). For PFOA, the corresponding coefficient was –0.006, *p* = 0.98. Although the meta-regression results support our hypothesis for PFOS, the lack of support for PFOA could be attributable to the small number of studies included, and other sources of heterogeneity.

In light of these results, epidemiologic studies investigating the effects of prenatal PFAS on fetal growth should account for the influence of GFR. Different approaches could be considered. An option would be to sample maternal plasma before pregnancy or during the first trimester, when changes in GFR have not yet influenced PFAS significantly according to simulated results. Statistically adjusting for GFR estimated from plasma creatinine levels or cystatin C levels (Tidman et al. 2008) could also help reduce confounding by GFR. Another approach would be to use a PBPK model to simulate results that are specific to their study sample collection time and PFAS distribution. Assuming the PBPK model and key assumptions are valid, the contribution of GFR to the observed association could be inferred from a comparison of simulated versus observed results. Two studies of communities with high exposure to PFOA have used PFOA serum levels estimated using one-compartment pharmacokinetic model coupled with a model for individual exposure to evaluate the association between prenatal exposure and birth outcomes (Savitz et al. 2012a, 2012b). Because the PFOA level estimates were not based on biological levels, the association between estimated levels and birth outcomes cannot be confounded by GFR. Of note, these studies were not suggestive of an association between prenatal PFOA exposure and birth weight (Savitz et al. 2012b). For example, in Savitz et al. (2012b), based on data for 4,534 births, the adjusted change in birth weight per 100-ng/mL increase in estimated serum PFOA was –15 g (95% CI: –43, 14).

Our results also have implications with regard to future meta-analyses of prenatal PFAS and birth weight. As noted by Egger et al. (1998), the real strength of meta-analyses is to identify factors responsible for heterogeneity across studies. According to our simulations, the contribution of GFR to the association between simulated PFAS levels and birth weight is influenced by the timing of sample collection and PFAS level distribution (mean and SD). A meta-analysis, including a meta-regression, based on more studies, and consideration of other sources of heterogeneity, would be of interest.

Certain assumptions might have introduced bias in our study. Because individual-specific data on GFR, PFAS, and birth weight were not available, we could evaluate the PBPK model validity only on a population level. Should extensive individual-specific measurements be available during pregnancy, the model could be further calibrated and evaluated. Nevertheless, when we simulated plasma PFAS levels across pregnancy in women from two studies who had their blood levels measured twice, simulated levels followed a decline in PFAS levels that closely matched reported levels. Because the simulated association between PFAS and birth weight was shown to be sensitive to the distribution of PFAS levels, the strength of the association between simulated PFAS levels and birth weight from this study cannot be compared with epidemiologic studies or meta-analyses with a different distribution of plasma PFAS levels. The coefficient of the GFR–birth weight association used in the Monte Carlo simulation was also shown to be a sensitive parameter. Should the true association between GFR and birth weight be stronger or weaker than the meta-analytic relation used in this study, one would expect the simulated association between PFAS and birth weight to change accordingly (i.e., a stronger GFR–birth weight association would increase the strength of the simulated PFAS–birth weight association and vice versa). We also did not account for the potential association between GFR and initial PFAS concentration at conception. For example, prepregnancy GFR was correlated with GFR during pregnancy in the Gibson (1973) study (*r* = 0.55–0.69) and in the Dunlop (1981) study (*r* = 0.27–0.30), although correlations were statistically significant only in the Gibson (1973) study. If prepregnancy GFR is associated with GFR during pregnancy, we could have underestimated the portion of the PFAS–birth weight association that is attributable to GFR by not accounting for the relationship between GFR and initial PFAS level. Also, we did not account for correlations across model parameters in Monte Carlo simulations, a factor that may have increased the spread of simulated blood PFAS levels (Burmaster and Anderson 1994). The assumptions that the initial plasma PFAS level is at steady state and that PFAS intake on a body weight basis is constant throughout pregnancy may oversimplify variations that are expected to occur in reality.

The meta-analysis for PFOA that we did was based on data for > 4,000 subjects. The more formal meta-analysis by Johnson et al. (2014) included two additional studies, each with < 50 subjects (Fromme et al. 2010; Kim S et al. 2011). In addition, the value we used to represent the data from the Washino et al. (2009) study was adjusted for more factors than was the one used by Johnson et al. (2014), and the value we used was closer to the null. Thus, the slightly more negative summary in Johnson et al. (2014) (–18.9 g/ng/mL) than in our study (–14.7 g/ng/mL) was probably attributable to the inclusion of the two additional studies and the different coefficient for the Washino et al. (2009) result. We regard the two meta-analyses as showing close agreement.

In a recent systematic review of the literature, Lam et al. (2014) concluded that there was sufficient evidence of an association between prenatal PFOA and fetal growth. Authors evaluated the hypothesis that GFR influences the PFOA–fetal growth association by reviewing the literature on GFR and fetal growth. They suggested that there is insufficient evidence for an association between maternal GFR during pregnancy and fetal growth, and they consequently rejected the hypothesis that GFR underlies the relationship between PFOA and fetal growth. However, Lam et al. (2014) did not include the study by Morken et al. (2014) in their systematic review of GFR and fetal growth, most likely because the results had not been published at the time. This new study by Morken et al. (2014), by far the largest to date (*n* = 953), revealed a significant association between estimated GFR and birth weight. When considering all available studies on the subject, we found that large studies consistently demonstrated an association between estimated GFR or indicators of GFR (e.g., serum creatinine, serum uric acid) and birth weight [Akahori et al. 2012 (*n* = 120); Knopp et al. 1985 (*n* = 272); Laughon et al. 2009 (*n* = 212); Morken et al. 2014 (*n* = 953)], whereas results from smaller studies have been inconsistent [Davison and Hytten 1974 (*n* = 10); Dunlop 1981 (*n* = 25); Dunlop et al. 1978 (*n* = 34); Duvekot et al. 1995 (*n* = 16); Gibson 1973 (*n* = 21)]. Given the new evidence, there is reason to believe a true association exists between maternal GFR during pregnancy and birth weight. Yet our results, which are based on the association between GFR and birth weight from three studies with individual-specific paired GFR and birth weight measurements (Dunlop 1981; Gibson 1973; Morken et al. 2014), are not in contrast with the conclusion of Lam et al. (2014). Rather than suggesting that GFR is the sole driver of the association between prenatal PFAS and birth weight, our results indicate that a portion of the association may be attributable to confounding by GFR, and that effect estimates may be overpredicted in epidemiologic studies where GFR is not accounted for.

## Conclusion

Results from our simulations suggest that epidemiologic studies of prenatal PFAS and birth weight may have overestimated the strength of the association. This study adds to existing studies demonstrating that pharmacokinetic models can be used to provide insight into the direction (Watkins et al. 2013) and the strength of epidemiologic associations (Verner et al. 2013). By combining results from epidemiologic studies with pharmacokinetic analyses, researchers will be able to identify underlying factors that can positively or negatively confound associations and to estimate their contribution to observed effect estimates.

## Supplemental Material

(767 KB) PDF
