# Supplementary material for: Associations of Perfluoroalkyl Substances (PFAS) with Lower Birth Weight: An Evaluation of Potential Confounding by Glomerular Filtration Rate Using a Physiologically Based Pharmacokinetic Model (PBPK)
Source: Environ Health Perspect. 2015 May 22;123(12):1317–24. doi: 10.1289/ehp.1408837 (PMC4671243; doi:10.1289/ehp.1408837)
Supplement: (767 KB) PDF [file ehp.1408837.s001.acco.pdf]

Environ Health Perspect
DOI: 10.1289/ehp.1408837

Note to Readers: *EHP* strives to ensure that all journal content is accessible to all readers. However, some figures and Supplemental Material published in *EHP* articles may not conform to 508 standards due to the complexity of the information being presented. If you need assistance accessing journal content, please contact ehp508@niehs.nih.gov. Our staff will work with you to assess and meet your accessibility needs within 3 working days.

Supplemental Material

Associations of Perfluoroalkyl Substances (PFASs) with Lower Birth Weight: An Evaluation of Potential Confounding by Glomerular Filtration Rate Using a Physiologically Based Pharmacokinetic Model (PBPK)

Marc-André Verner, Anne E. Loccisano, Nils-Halvdan Morken, Miyoung Yoon, Huali Wu, Robin McDougall, Mildred Maisonet, Michele Marcus, Reiko Kishi, Chihiro Miyashita, Mei-Huei Chen, Wu-Shiun Hsieh, Melvin E. Andersen, Harvey J. Clewell III, and Matthew P. Longnecker

Table of Contents

Figure S1. Conceptual representation of the PBPK model (Loccisano et al. 2013) including mass-balance differential equations. The different symbols are spelled out on next page.
Symbols
Table S1. List of studies included or excluded from meta-analysis of birth weight and PFAS, with a brief description of each.
Table S2. Sensitivity coefficients for the most sensitive model parameters.
PBPK Model Code
References

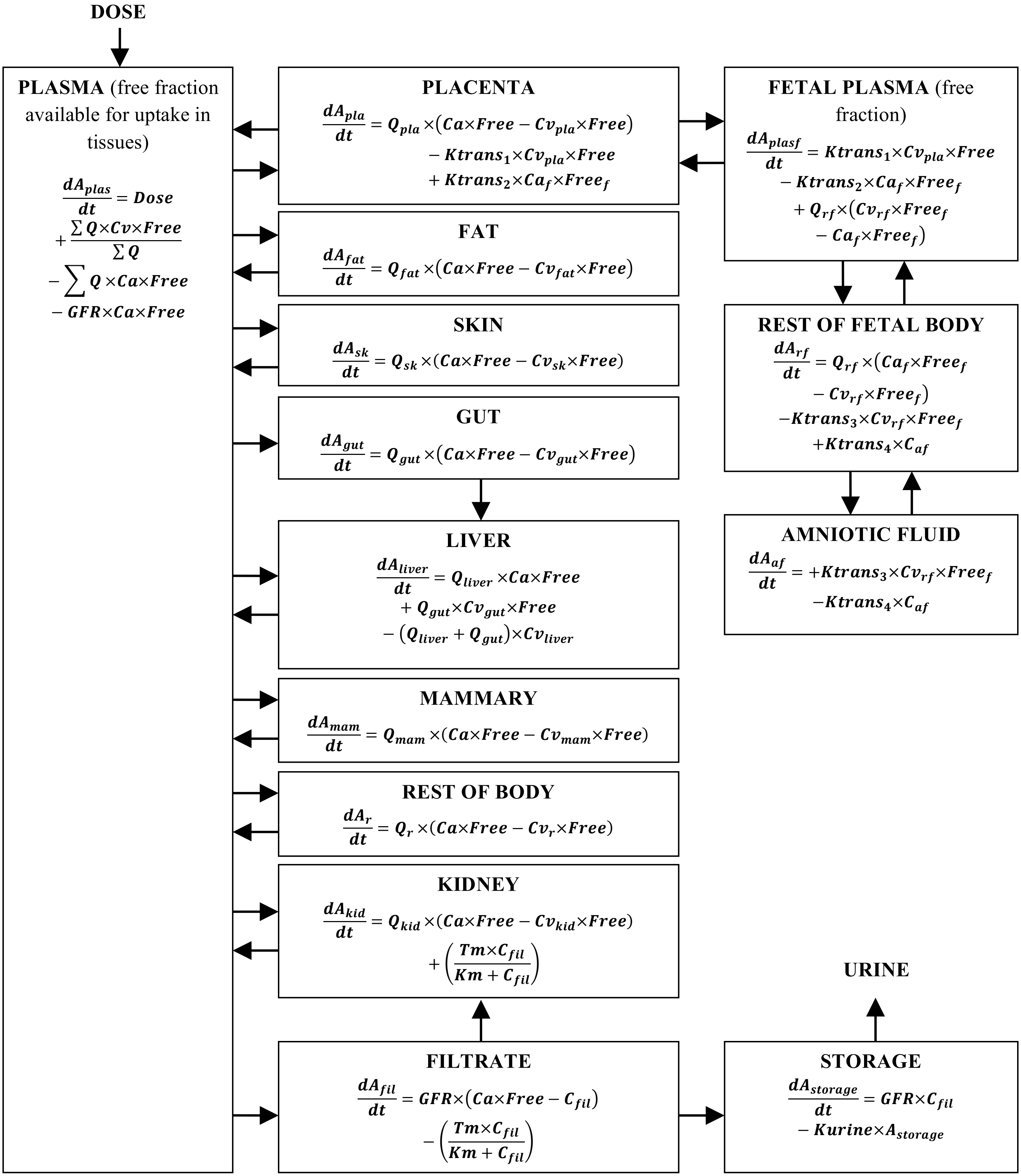

Figure S1. Conceptual representation of the PBPK model (Loccisano et al. 2013) including mass-balance differential equations. The different symbols are spelled out on next page.

Symbols

A_{af}	Amount in amniotic fluid
A_{fat}	Amount in fat
A_{fil}	Amount in filtrate
A_{gut}	Amount in gut
A_{kid}	Amount in kidneys
A_{liver}	Amount in liver
A_{mam}	Amount in mammary tissue
A_{pla}	Amount in placenta
A_{plas}	Amount in plasma (mother)
A_{plasf}	Amount in plasma (fetus)
A_{r}	Amount in rest of body (mother)
A_{rf}	Amount in rest of body (fetus)
A_{sk}	Amount in skin
$A_{storage}$	Amount in storage compartment
Ca	Arterial plasma concentration (mother)
Ca_{f}	Arterial plasma concentration (foetus)
C_{af}	Concentration in amniotic fluid
C_{fil}	Concentration in filtrate
Cv	Concentration in total venous plasma
Cv_{fat}	Concentration in venous plasma from the fat
Cv_{gut}	Concentration in venous plasma from the gut
Cv_{kid}	Concentration in venous plasma from the kidneys
Cv_{liver}	Concentration in venous plasma from the liver
Cv_{mam}	Concentration in venous plasma from the mammary tissue
Cv_{pla}	Concentration in venous plasma from the placenta
Cv_{r}	Concentration in venous plasma from the rest of body (mother)
Cv_{rf}	Concentration in venous plasma from the rest of body (fetus)
Cv_{sk}	Concentration in venous plasma from skin
Free	Free fraction in plasma (mother)
$Free_{f}$	Free fraction in plasma (fetus)
GFR	Glomerular filtration rate
Km	Renal transporter affinity constant
Ktrans1	Placental diffusion constant (from mother to fetus)
Ktrans2	Placental diffusion constant (from fetus to mother)
Ktrans3	Transfer constant (from fetus to amniotic fluid)
Ktrans4	Transfer constant (from amniotic fluid to fetus)
Kurine	Urinary elimination rate constant
Q	Plasma perfusion rate
Q_{fat}	Plasma perfusion rate to the fat
Q_{gut}	Plasma perfusion rate to the gut
Q_{kid}	Plasma perfusion rate to the kidneys
Q_{liver}	Plasma perfusion rate to the liver
Q_{mam}	Plasma perfusion rate to the mammary tissue
Q_{pla}	Plasma perfusion rate to the placenta
Q_{r}	Plasma perfusion rate to the rest of body (mother)
Q_{rf}	Plasma perfusion rate to the rest of body (fetus)
Q_{sk}	Plasma perfusion rate to the skin
Tm	Renal transporter maximum resorption velocity

Table S1. List of studies included or excluded from meta-analysis of birth weight and PFAS, with a brief description of each.

Source	Country	Period	N	Comments
Included				
Apelberg et al. 2007	U.S.A.	2004-2005	293	
Fei et al. 2007	Denmark	1996-2002	1,399	
Hamm et al. 2009	Canada	2005-2006	252	
Washino et al. 2009	Japan	2002-2005	428	
Chen et al. 2012	Taiwan	2004-2005	429	
Whitworth et al. 2012	Norway	2003-2004	849	
Maisonet et al. 2012	Great Britain	1991-1992	422	
Excluded				
Inoue et al. 2004	Japan	2003	15	Univariate. Birth weight was the independent variable.
Monroy et al. 2008	Canada	2004-2005	89	Univariate. Birth weight was the independent variable.
Nolan, 2009	U.S.A.	2003-2005	1,555	Univariate. Exposure was based on water district.
Stein et al. 2009	U.S.A.	2005-2006	1,589-4,561	PFAS measured after pregnancy. Low birth weight was the outcome.
Kim et al. 2011	Korea	2008-2009	44	Regression coefficient not presented.
Savitz et al. 2012a	U.S.A.	2005-2006	11,737	PFAS estimated from model. Low birth weight was the outcome.
Savitz et al. 2012b	U.S.A.	1990-2004	8,253	PFAS estimated from model.
Halldorrson et al. 2012	Denmark	1988-1989	655	Univariate
Wu et al. 2012	China	2007	158	See footnote

We conducted in the Pubmed search in 2012 and after the manuscript for this article was provisionally accepted we repeated the search as a check. The search in April of 2015 revealed that the Wu et al. (2012) article also met the inclusion criterion, and had data on PFOA. When we repeated the meta-analysis for PFOA including the Wu et al. (2012) results, the summary coefficient from a random effects models was -12.4 g birth weight (SE 3.7), which was close to our original results (-14.7 g). To calculate this estimate, however, we assumed that a $\log_{10}$ increase in PFOA was equivalent to 50 ng/ml, and we were not confident about this re-expression. Thus, we did not update the meta-analysis. We also repeated the meta-regression for PFOA, and with the data from Wu et al. (2012) included, the coefficient for week of blood draw was not statistically significant, as before.

Table S2. Sensitivity coefficients for the most sensitive model parameters.

	Initial body weight	Liver volume as a fraction of body weight	Liver: plasma partition coefficient	Rest of body:plasma partition coefficient	Free fraction in maternal plasma	Free fraction in fetal plasma	Resorption maximum velocity	Affinity constant	Standardized glomerular filtration rate (GFR_{ratio})
PFOS									
Maternal plasma									
1st month	0.00025482	0.00004976	0.00004027	0.00004649	0.00013986	0.00000000	0.00010732	0.00013918	0.00027270
2nd month	0.00055318	0.00010385	0.00008401	0.00009799	0.00030334	0.00000027	0.00023395	0.00029977	0.00057214
3rd month	0.00087194	0.00017493	0.00014362	0.00017316	0.00047710	0.00000274	0.00036859	0.00046620	0.00089098
4th month	0.00125927	0.00027143	0.00022592	0.00028111	0.00068930	0.00001233	0.00053090	0.00066052	0.00126904
5th month	0.00172690	0.00039098	0.00032767	0.00041777	0.00095053	0.00003680	0.00072525	0.00088372	0.00171160
6th month	0.00210177	0.00048521	0.00040702	0.00052628	0.00116418	0.00006802	0.00088081	0.00105471	0.00205999
7th month	0.00242076	0.00056382	0.00047244	0.00061720	0.00134951	0.00010523	0.00101352	0.00119526	0.00235614
8th month	0.00268585	0.00062637	0.00052392	0.00069034	0.00150833	0.00015136	0.00112232	0.00130701	0.00259492
Delivery	0.00292021	0.00068096	0.00056859	0.00075549	0.00165564	0.00021251	0.00121457	0.00139978	0.00279755
Cord plasma									
Delivery	0.00188033	0.00032544	0.00063431	0.00051174	0.00269457	0.00457312	0.00078186	0.00095558	0.00249216
PFOA									
Maternal plasma									
1st month	0.00008960	0.00002928	0.00002016	0.00002822	0.00006194	0.00000000	0.00004355	0.00004470	0.00010143
2nd month	0.00019174	0.00005798	0.00004023	0.00005571	0.00012973	0.00000007	0.00009355	0.00009600	0.00020815
3rd month	0.00029740	0.00008806	0.00006425	0.00008698	0.00019617	0.00000068	0.00014487	0.00014854	0.00031847
4th month	0.00042126	0.00012149	0.00009465	0.00012450	0.00026942	0.00000292	0.00020422	0.00020918	0.00044361
5th month	0.00056227	0.00015499	0.00013065	0.00016572	0.00034664	0.00000811	0.00027093	0.00027728	0.00058004
6th month	0.00066513	0.00017537	0.00015760	0.00019371	0.00039773	0.00001411	0.00031994	0.00032729	0.00067768
7th month	0.00074250	0.00018789	0.00017846	0.00021318	0.00043214	0.00002059	0.00035806	0.00036608	0.00075302
8th month	0.00079924	0.00019460	0.00019334	0.00022552	0.00045529	0.00002840	0.00038728	0.00039585	0.00080887
Delivery	0.00084311	0.00019813	0.00020495	0.00023402	0.00047216	0.00003864	0.00041043	0.00041948	0.00085270
Cord plasma									
Delivery	0.00091908	0.00026732	0.00032616	0.00026743	0.00115986	0.00147319	0.00056451	0.00044270	0.00111788

Sensitivity coefficients were calculated by adapting the M code of the Morris Test included in the acslX Optimum suite of tools (Aegis Technologies Inc., Huntsville, AL, USA) to our study.

```
! MODEL CODE FOR ACSLX (AEGIS TECHNOLOGIES GROUP, INC, HUNSTVILLE, AL, USA)

PROGRAM PFAS_GESTATION_MC

!----------------------------------------------!
! PBPK model for PFOS/PFOA in pregnant human   !
! Coded by Anne Loccisano                      !
! Modified by Marc-Andre Verner                !
!                                              !
! Units                                        !
! Amounts: mg                                  !
! Volumes: L                                   !
! Time: h                                      !
!----------------------------------------------!

INITIAL

!-----------------------------------------------------------------------------------------------
! Compound (Only one compound can have a value of "1")
constant PFOS = 1    ! PFOS? (1:Yes, 0:No)
constant PFOA = 0    ! PFOA? (1:Yes, 0:No)

!-----------------------------------------------------------------------------------------------
! Initial venous blood concentration (mg/l or ug/ml)
constant CVINIT = 0.013

!-----------------------------------------------------------------------------------------------
! Simulation time in hours (9 months = 6570 h)
constant TSTOP = 6570

!-----------------------------------------------------------------------------------------------
! Constant physiological parameters

! Body weight
constant BWINIT = 60.9        ! Pre-pregnancy body weight (kg)

! Maternal blood flows
constant QCC    = 20.0        ! Cardiac blood output (L/h/kg**0.75)
constant QLC    = 0.065       ! Fraction cardiac output going to liver through hepatic artery
constant QSkC   = 0.058       ! Fraction of cardiac output going to skin
constant QGC    = 0.181       ! Fraction of cardiac output going to gut
constant QMamC  = 0.027       ! Initial fraction of cardiac output going to mammary tissue
constant QFatC  = 0.052       ! Initial fraction of cardiac output going to fat
```

```
TABLE DQK,1,26/0,8,10,11,12,13,15,17,18,19,22,23,24,25,26,27,28,29,31,32,33,34,36,37,38,40, &
24.42,50.58,45.42,51.36,57.12,46.32,44.73,45.36,41.8,46.53,46.08,47.85,43.05,41.16,47.52,43.41,&
48.57,46.36,36.68,39.44,46.46,44.13,26.7,33.92,35.46,40.26/  ! Renal plasma flow (L/h) [INDEX:WK]
TABLE DQFil,1,40/0,1,2,3,4,5,6,7,8,9,10,11,12,13,14,15,16,17,18,19,20,21,22,23,24,25,26,27,28, &
29,30,31,32,33,34,35,36,37,38,39,6.0,6.0,6.0,6.0,6.0,6.03,6.09,6.18,6.32,6.50,6.72,6.97,7.23, &
7.50,7.76,8.01,8.23,8.43,8.60,8.75,8.86,8.95,9.01,9.05,9.06,9.06,9.04,8.99,8.95,8.88,8.81,8.72, &
8.62,8.52,8.41,8.29,8.16,8.03,7.89,7.75/   ! Glomerular filtration rate (L/h) [INDEX:WK]

! Maternal tissue volumes
constant VLC      = 0.026    ! Liver volume as a fraction of initial BW
constant VKC      = 0.004    ! Kidney volume as a fraction of initial BW
constant VfilC    = 0.0004   ! Filtrate compartment volume as a fraction of initial BW
constant VGC      = 0.0171   ! Gut volume as a fraction of initial BW
constant VMamC    = 0.0062   ! Mammary tissue volume as a fraction of initial BW
constant VFatC    = 0.214    ! Fat volume as a fraction of initial BW
constant VSk      = 0.0972   ! Skin volume (L)
TABLE DVPlasC,1,28/9,10,11,12,13,14,15,16,17,18,19,20,21,22,23,24,25,26,27,28,29,30,31,32,&
33,34,35,36,0.0442,0.0445,0.0448,0.0451,0.0454,0.0456,0.0458,0.0460,0.0461,0.0462,0.0463, &
0.0464,0.0464,0.0464,0.0464,0.0463,0.0463,0.0461,0.0460,0.0458,0.0456,0.0454,0.0451,0.0448, &
0.0445,0.0441,0.0437,0.0433/     ! Maternal plasma volume as a fraction of BW [INDEX:WK]
TABLE DHtc,1,9/0,11,16,20,24,28,32,36,40,0.38,0.372,0.356,0.354,0.349,0.347, &
0.346,0.354,0.367/     ! Hematocrit [INDEX:WK]
TABLE VAFX,1,20/0,8,9,10,11,12,16,18,20,22,24,26,28,30,32,34,36,38,40,42,0.0237,0.0235,&
0.0234,0.0232,0.0766,0.124,0.195,0.284,0.361,0.646,0.640,0.687,0.770,0.824,0.776,0.817, &
0.817,0.799,0.530,0.506/     ! Amniotic fluid volume (L) [INDEX:WK]

!-----------------------------------------------------------------------------------------------
! Chemical specific parameters

! Tissue:plasma partition coefficients (from rat)
constant PFOS_PL   = 3.72     ! PFOS Liver:plasma partition coefficient
constant PFOS_PFat = 0.14     ! PFOS Fat:plasma partition coefficient
constant PFOS_PK   = 0.80     ! PFOS Kidney:plasma partition coefficient
constant PFOS_PSk  = 0.29     ! PFOS Skin:plasma partition coefficient
constant PFOS_PR   = 0.20     ! PFOS Rest of body:plasma partition coefficient
constant PFOS_PG   = 0.57     ! PFOS Gut:plasma partition coefficient
constant PFOS_PMam = 0.16     ! PFOS Mammary tissue:plasma partition coefficient
constant PFOS_PPla = 0.41     ! PFOS Placenta:plasma partition coefficient
constant PFOS_PRF  = 0.20     ! PFOS Rest of fetal body:plasma partition coefficient
constant PFOA_PL   = 2.2      ! PFOA Liver:plasma partition coefficient
constant PFOA_PFat = 0.04     ! PFOA Fat:plasma partition coefficient
constant PFOA_PK   = 1.05     ! PFOA Kidney:plasma partition coefficient
constant PFOA_PSk  = 0.1      ! PFOA Skin:plasma partition coefficient
```

```
constant PFOA_PR   = 0.12     ! PFOA Rest of body:plasma partition coefficient
constant PFOA_PG   = 0.05     ! PFOA Gut:plasma partition coefficient
constant PFOA_PMam = 0.13     ! PFOA Mammary tissue:plasma partition coefficient
constant PFOA_PPla = 0.28     ! PFOA Placenta:plasma partition coefficient
constant PFOA_PRF  = 0.12     ! PFOA Rest of fetal body:plasma partition coefficient

PL   = PFOS*PFOS_PL   + PFOA*PFOA_PL     ! Liver:plasma partition coefficient
PFat = PFOS*PFOS_PFat + PFOA*PFOA_PFat   ! Fat:plasma partition coefficient
PK   = PFOS*PFOS_PK   + PFOA*PFOA_PK     ! Kidney:plasma partition coefficient
PSk  = PFOS*PFOS_PSk  + PFOA*PFOA_PSk    ! Skin:plasma partition coefficient
PR   = PFOS*PFOS_PR   + PFOA*PFOA_PR     ! Rest of the body:plasma partition coefficient
PG   = PFOS*PFOS_PG   + PFOA*PFOA_PG     ! Gut:plasma partition coefficient
PMam = PFOS*PFOS_PMam + PFOA*PFOA_PMam   ! Mammary:plasma partition coefficient
PPla = PFOS*PFOS_PPla + PFOA*PFOA_PPla   ! Placenta:plasma partition coefficient
PRF  = PFOS*PFOS_PRF  + PFOA*PFOA_PRF    ! Rest of fetal body:plasma partition coefficient

! Renal reabsorption
constant PFOS_TMC = 3.5                  ! Reabsorption maximum PFOS (mg/h/kg**0.75)
constant PFOA_TMC = 10.0                 ! Reabsorption maximum PFOA (mg/h/kg**0.75)
TMC = PFOS*PFOS_TMC + PFOA*PFOA_TMC      ! Reabsorption maximum (mg/h/kg**0.75)
constant PFOS_KT = 0.023                 ! Affinity constant PFOS (mg/L)
constant PFOA_KT = 0.055                 ! Affinity constant PFOA (mg/L)
KT  = PFOS*PFOS_KT + PFOA*PFOA_KT        ! Affinity constant (mg/L)

! Binding constants
constant PFOS_Free  = 0.025              ! Free fraction of PFOS in maternal plasma
constant PFOA_Free  = 0.020              ! Free fraction of PFOA in maternal plasma
Free  = PFOS*PFOS_Free+PFOA*PFOA_Free    ! Free fraction in maternal plasma
constant PFOS_FreeF = 0.025              ! Free fraction in fetal plasma PFOS
constant PFOA_FreeF = 0.020              ! Free fraction in fetal plasma PFOA
FreeF = PFOS*PFOS_FreeF+PFOA*PFOA_FreeF  ! Free fraction in fetal plasma

! Urinary excretion
constant kurinec = 0.005      ! Urinary elimination rate constant (/h/kg**-0.25)

! Placental transfer
constant PFOS_k1c = 0.46                    ! Mom to fetus PFOS (L/h/kg**0.75)
constant PFOA_k1c = 0.46                    ! Mom to fetus PFOA (L/h/kg**0.75)
ktrans1c = PFOS*PFOS_k1c + PFOA*PFOA_k1c    ! Mom to fetus (L/h/kg**0.75)
constant PFOS_k2c = 1.01                    ! Fetus to mom PFOS (L/h/kg**0.75)
constant PFOA_k2c = 0.46                    ! Fetus to mom PFOA (L/h/kg**0.75)
ktrans2c = PFOS*PFOS_k2c + PFOA*PFOA_k2c    ! Fetus to mom (L/h/kg**0.75)
```

```
! Amniotic fluid transfer
constant PFOS_k3c = 0.006          ! Amniotic fluid to fetus PFOS (L/h/kg**0.75)
constant PFOA_k3c = 0.008          ! Amniotic fluid to fetus PFOA (L/h/kg**0.75)
ktrans3c = PFOS*0.006+ PFOA*0.008  ! Amniotic fluid to fetus (L/h/kg**0.75)
constant PFOS_k4c = 0.001          ! Fetus to fluid PFOS (L/h/kg**0.75)
constant PFOA_k4c = 0.001          ! Fetus to fluid PFOA (L/h/kg**0.75)
ktrans4c = PFOS*0.001+PFOA*0.001   ! Fetus to fluid (L/h/kg**0.75)

END ! INITIAL

DYNAMIC ! Start dynamic section

ALGORITHM IALG = 15        ! Use CVODE algorithm
CINTERVAL CINT = 100       ! Communication interval
MINTERVAL MINT = 10e-09    ! Minimum interval
MAXTERVAL MAXT = 1.0       ! Maximum interval

DERIVATIVE

!-------------------------------------------------------------------------------------------------
! Timing parameters
GD = T/24     ! Gestation day
WK = GD/7     ! Gestation week

!-------------------------------------------------------------------------------------------------
! Organ volume calculation

! Fetal growth
VFet_av = (3.779*exp(-16.081*(exp(-5.67e-4*(GD*24)))) &
         + 3.833*exp(-140.178*(exp(-7.01e-4*(GD*24)))))  ! Average fetal volume (L)
constant Birthweight = 3.50857                           ! Birth weight (kg)
VFet                 = (Birthweight/3.50857)*VFet_av     ! Adjustment of fetal growth (L)

! Amniotic fluid
constant AF_VAF = 1.0      ! Adjustment factor for amniotic fluid
VAF = AF_VAF*VAFX(WK)      ! Amniotic fluid volume (L)

! Hematocrit
constant HtcF   = 0.5     ! Fetal hematocrit
constant AF_Htc = 1.0     ! Adjustment factor for maternal hematocrit
HtcINIT = AF_Htc*DHtc(0)  ! Initial maternal hematocrit
Htc     = AF_Htc*DHtc(WK) ! Maternal hematocrit
```

```
! Plasma volumes
constant VPlasFC   = 0.0428              ! Fetal plasma volume as a fraction of fetal weight
constant AF_VPlasF = 1.0                 ! Adjustment factor for fetal plasma volume
VPlasF    = AF_VPlasF*VPlasFC*VFet       ! Fetal plasma volume (L)
constant AF_VPlas = 1.0                  ! Adjustment factor for maternal plasma volume
VPlasINIT = AF_VPlas*DVPlasC(0)*BWINIT   ! Initial plasma volume (L)
VPlas     = AF_VPlas*DVPlasC(WK)*BW      ! Maternal plasma volume (L)

! Placenta volume
constant AF_VPla = 1.0                                  ! Adjustment factor for placenta volume
VPla_av  = (0.85*exp(-9.434*(exp(-5.23e-4*(GD*24))))) ! Average placenta volume (L)
Vplainit = AF_VPla*0.85*exp(-9.434*(exp(0)))           ! Initial placenta volume (L)
VPla     = AF_VPla*VPla_av                              ! Placenta volume (L)

! Other tissue volumes
VL      = VLC*BWINIT                       ! Liver volume (L)
VK      = VKC*BWINIT                       ! Kidney volume (L)
Vfil    = VfilC*BWINIT                     ! Fitrate compartment volume (L)
VG      = VGC*BWINIT                       ! Gut volume (L)
VMamINIT = VMamC*BWINIT                    ! Initial mammary volume (L)
VFatINIT = VFatC*BWINIT                    ! Initial fat volume (L)
VR      = (0.84*BWINIT)-VL-VK-VFil &
         -VG-VPlasINIT-VSk-VMamINIT &
         -VFatINIT                         ! Rest of maternal body volume (L)
VRF     = (0.92*VFet)-VPlasF               ! Volume of rest of fetal body (L)

VMam    = BWINIT*(((VMamC+(0.0065*exp(-7.444868477* &
          (exp(-0.000678*(GD*24))))))))               ! Mammary tissue volume (L)
VFat    = BWINIT*(((VFatC+(0.09*exp(-12.90995862* &
          (exp(-0.000797*(GD*24))))))))               ! Fat volume (L)
BW      = BWINIT+(VFat-VFATINIT)+(VMam-VMamINIT) &
          +VPla+VFet+VAF                               ! Body weight (kg)

!---------------------------------------------------------------------------------------------------
! Blood flow calculation

! Fetal cardiac output
constant QFetC      = 24.0       ! Fetal cardiac blood output (L/h/kg fetal BW)
QFet = QFetC*VFet*(1-HtcF)       ! Fetal cardiac plasma output using fetal hematocrit (L/h)
QRF  = QFet                      ! Flow to rest of body (L/h)

! Other flows
QCINIT   = QCC*BWINIT**0.75                            ! Initial cardiac blood output (L/h)
```

```
QCPINIT  = QCINIT*(1-HtcINIT)                          ! Initial cardiac plasma output (L/h)
QL       = QLC*QCPINIT                                 ! Plasma flow to liver (L/h)
QG       = QGC*QCPINIT                                 ! Plasma flow to gut (L/h)
QSk      = QSkC*QCPINIT                                ! Plasma flow to skin (L/h)
QFatINIT = QFatC*QCPINIT                               ! Initial flow to fat
QFat     = QFatINIT*(VFat/VFatINIT)                    ! Plasma flow to fat (L/h)
QMamINIT = QMamC*QCPinit                               ! Intial flow to mammary tissue
QMam     = QMamINIT*(VMam/VMamINIT)                    ! Plasma flow to mammary tissue (L/h)
QC       = QCPINIT+(QFat-QFatINIT)+(QMam-QMamINIT) &
          +(QK-QKINIT)+ (QFil-QFilINIT)+ QPla          ! Total cardiac plasma output (L/h)
QR       = QC-(QL+QK+QG+QSk+QFat+QMam+QPla)            ! Plasma flow to rest of body (L/h)
Qbal     = QC-(QL+QK+QG+QSk+QFat+QMam+QPla+QR)         ! Balance check
kurine   = kurinec*(BWINIT**(-0.25))                   ! Urine elimination from urine storage
Tm       = Tmc*BW**0.75                                ! Transporter maximum

! Plasma flow to placenta
constant QPlaC = 0.33       ! Plasma flow to placenta as a fraction of fetal cardiac output
QPla = QPlaC*QFet           ! Plasma flow to placenta (L/h)

! Plasma flow to kidney
constant AF_QK = 1.0        ! Adjustment factor for plasma flow to kidney
QKINIT  = AF_QK*DQK(0)      ! Initial plasma flow to kidney
QK      = AF_QK*DQK(WK)     ! Plasma flow to kidney (l/h)

! Glomerular filtration rate
constant Ratio_GFR    = 1.0         ! Adjustment factor for GFR
QFil_R   = DQFil(WK)                ! Average GFR (L/h)
QFilINIT = Ratio_GFR*DQFil(0)       ! Initial GFR (L/h)
QFil     = Ratio_GFR*QFil_R         ! GFR (L/h)

! Placental & amniotic fluid transfer
ktrans1 = ktrans1c*(VFet**0.75)     ! Transfer from placenta to fetal plasma (L/h)
ktrans2 = ktrans2c*(VFet**0.75)     ! Transfer from fetal plasma to placenta (L/h)
ktrans3 = ktrans3c*(VFet**0.75)     ! Transfer from rest of fetal body to amniotic fluid (L/h)
ktrans4 = ktrans4c*(VFet**0.75)     ! Transfer from amniotic fluid to rest of fetal body (L/h)
```

```
!---------------------------------------------------------------------------------
! Sampling times (collection of blood levels)
constant First_sample_t   = 2500    ! Timing of first sample (h)
constant Second_sample_t  = 6569    ! Timing of second sample (h)

! Setting initial values for blood concentrations
! *Flag values 99999 are replaced by simulated levels at recording times
initial
CV_SAMPLE1   = 99999    ! Blood level in 1st sample before it is recorded
CV_SAMPLE2   = 99999    ! Blood level in 2nd sample before it is recorded
CV_SAMPLE_M1 = 99999    ! Blood level at 1st month of pregnancy before it is recorded
CV_SAMPLE_M2 = 99999    ! Blood level at 2nd month of pregnancy before it is recorded
CV_SAMPLE_M3 = 99999    ! Blood level at 3rd month of pregnancy before it is recorded
CV_SAMPLE_M4 = 99999    ! Blood level at 4th month of pregnancy before it is recorded
CV_SAMPLE_M5 = 99999    ! Blood level at 5th month of pregnancy before it is recorded
CV_SAMPLE_M6 = 99999    ! Blood level at 6th month of pregnancy before it is recorded
CV_SAMPLE_M7 = 99999    ! Blood level at 7th month of pregnancy before it is recorded
CV_SAMPLE_M8 = 99999    ! Blood level at 8th month of pregnancy before it is recorded
CV_SAMPLE_M9 = 99999    ! Blood level at 9th month of pregnancy before it is recorded
CORD_SAMPLE  = 99999    ! Cord blood level before it is recorded
end

! Record blood concentration at sampling times
schedule blood_samp1 .xn. (First_sample_t-t)  ! At 1st sampling time, blood_samp1 is "true"
discrete blood_samp1                          ! When blood_samp1 is "true"...
CV_SAMPLE1 = Ca                               ! Record total maternal plasma level
end
schedule blood_samp2 .xn. (Second_sample_t-t) ! At 2nd sampling time, blood_samp2 is "true"
discrete blood_samp2                          ! When blood_samp2 is "true"...
CV_SAMPLE2  = Ca                              ! Record total maternal plasma level
end
schedule blood_samp_m1 .xn. (730-t)           ! At 1st month of pregnancy, blood_samp_m1 is "true"
discrete blood_samp_m1                        ! When blood_samp_m1 is "true"...
CV_SAMPLE_M1  = Ca                            ! Record total maternal plasma level
end
schedule blood_samp_m2 .xn. (1460-t)          ! At 2nd month of pregnancy, blood_samp_m2 is "true"
discrete blood_samp_m2                        ! When blood_samp_m2 is "true"...
CV_SAMPLE_M2  = Ca                            ! Record total maternal plasma level
end
schedule blood_samp_m3 .xn. (2190-t)          ! At 3rd month of pregnancy, blood_samp_m3 is "true"
discrete blood_samp_m3                        ! When blood_samp_m3 is "true"...
CV_SAMPLE_M3  = Ca                            ! Record total maternal plasma level
end
```

```
schedule blood_samp_m4 .xn. (2920-t)          ! At 4th month of pregnancy, blood_samp_m4 is "true"
discrete blood_samp_m4                        ! When blood_samp_m4 is "true"...
CV_SAMPLE_M4  = Ca                            ! Record total maternal plasma level
end
schedule blood_samp_m5 .xn. (3650-t)          ! At 5th month of pregnancy, blood_samp_m5 is "true"
discrete blood_samp_m5                        ! When blood_samp_m5 is "true"...
CV_SAMPLE_M5  = Ca                            ! Record total maternal plasma level
end
schedule blood_samp_m6 .xn. (4380-t)          ! At 6th month of pregnancy, blood_samp_m6 is "true"
discrete blood_samp_m6                        ! When blood_samp_m6 is "true"...
CV_SAMPLE_M6  = Ca                            ! Record total maternal plasma level
end
schedule blood_samp_m7 .xn. (5110-t)          ! At 7th month of pregnancy, blood_samp_m7 is "true"
discrete blood_samp_m7                        ! When blood_samp_m7 is "true"...
CV_SAMPLE_M7  = Ca                            ! Record total maternal plasma level
end
schedule blood_samp_m8 .xn. (5840-t)          ! At 8th month of pregnancy, blood_samp_m8 is "true"
discrete blood_samp_m8                        ! When blood_samp_m8 is "true"...
CV_SAMPLE_M8  = Ca                            ! Record total maternal plasma level
end
schedule blood_samp_m9 .xn. (6569-t)          ! At 9th month of pregnancy, blood_samp_m9 is "true"
discrete blood_samp_m9                        ! When blood_samp_m9 is "true"...
CV_SAMPLE_M9  = Ca                            ! Record total maternal plasma level
CORD_SAMPLE   = Ca_f                          ! Record total cord plasma level
end

!-------------------------------------------------------------------------------------------------
! Dosing parameters
Vol_dist = VPLASINIT+PG*VG+PL*VL &
           +PFat*VFATINIT+PK*VK+PR*VR &
           +PMam*VMamINIT+PSk*VSk                              ! Volume of distribution (L)
constant DOSE_INT = 1                                          ! Dose interval (h)
constant PFOS_HL  = 5.4                                        ! Half-life PFOS (years)
constant PFOA_HL  = 3.8                                        ! Half-life PFOA (years)
HALF_LIFE         = (PFOS*PFOS_HL+PFOA*PFOA_HL)*365*24         ! Half-life (h)
IVHOURLYDOSE      = CVINIT*Vol_dist*DOSE_INT/(1.44*HALF_LIFE)  ! Hourly dose (mg/h)

!-------------------------------------------------------------------------------------------------
! Initial amounts of PFOS/PFOA in tissues (from initial blood level)
APLAS0  = CVINIT*VPLASINIT*Free    ! Amount free in plasma (mg)
AG0     = CVINIT*PG*VG             ! Amount in gut (mg)
AL0     = CVINIT*PL*VL             ! Amount in liver (mg)
AFAT0   = CVINIT*PFat*VFATINIT     ! Amount in fat (mg)
```

```
AK0     = CVINIT*PK*VK              ! Amount in kidney (mg)
AR0     = CVINIT*PR*VR              ! Amount in rest of body (mg)
AMAM0   = CVINIT*PMam*VMamINIT      ! Amount in mammary tissue (mg)
ASK0    = CVINIT*PSk*VSk            ! Amount in skin (mg)


!-------------------------------------------------------------------------------------------------
! MASS BALANCE DIFFERENTIAL EQUATIONS

!-- MOTHER --!

! FAT
RFat  = QFat*(CA*Free-CVFat*Free)   ! Rate of amount in fat (mg/h)
AFat  = integ(RFat,AFat0)           ! Amount in fat (mg)
CFat  = AFat/VFat                   ! Concentration in fat (mg/L)
CVFat = CFat/PFat                   ! Fat venous blood concentration (mg/L)

! MAMMARY TISSUE
RMam  = QMam*(CA*Free-CVMam*Free)   ! Rate of amount in mammary tissue (mg/h)
AMam  = integ(RMam,AMam0)           ! Amount in mammary tissue (mg)
CMam  = AMam/VMam                   ! Concentration in mammary tissue (mg/L)
CVMam = CMam/PMam                   ! Mammary tissue venous blood concentration (mg/L)

! GUT
RG  = QG*(CA*Free-CVG*Free)         ! Rate of amount in gut (mg/h)
AG  = integ(RG,AG0)                 ! Amount in gut (mg)
CG  = AG/VG                         ! Concentration in gut (mg/L)
CVG = CG/PG                         ! Gut venous blood concentration (mg/L)

! LIVER
RL  = (QL*(CA*Free)) &
      +(QG*CVG*Free) &
      -((QL+QG)*CVL*Free)           ! Rate of amount in liver (mg/h)
AL  = integ(RL,AL0)                 ! Amount in liver (mg)
CL  = AL/VL                         ! Concentration in liver (mg/L)
CVL = CL/PL                         ! Liver venous blood concentration (mg/L)

! KIDNEY
RK  = QK*(CA*Free-CVK*Free) &
      +(Tm*Cfil)/(Kt+Cfil)          ! Rate of amount in kidney (mg/h)
AK  = integ(RK,AK0)                 ! Amount in kidney (mg)
CK  = AK/VK                         ! Concentration in kidney (mg/L)
CVK = CK/PK                         ! Kidney venous blood concentration (mg/L)
```

```
! FILTRATE COMPARTMENT
RFil = Qfil*(CA*Free-Cfil) &
       -(Tm*Cfil)/(Kt+Cfil)         ! Rate of amount in filtrate compartment (mg/h)
AFil = integ(RFil,0.0)              ! Amount in filtrate compartment (mg)
Cfil = Afil/Vfil                    ! Concentration in filtrate compartment (mg/L)

! "DELAY" compartment for urine
Rdelay = Qfil*Cfil-kurine*Adelay    ! Rate of amount in "delay" compartment (mg/h)
Adelay = integ(Rdelay,0.0)          ! Amount in "delay" compartment (mg)

! URINE
RUrine = kurine*Adelay              ! Rate of amount excreted in urine (mg/h)
Aurine = integ(Rurine,0.0)          ! Amount excreted in urine (mg)

! SKIN
RSk  = QSk*(CA*Free-CVSk*Free)      ! Rate of amoune in skin (mg/h)
ASk  = integ(RSk,Ask0)              ! Amount in skin (mg)
CSk  = ASk/VSk                      ! Concentration in skin (mg/L)
CVSk = CSk/PSk                      ! Skin venous blood concentration (mg/L)

! REST OF BODY
RR  = QR*(CA*Free-CVR*Free)         ! Rate of amount in rest of body (mg/h)
AR  = integ(RR,AR0)                 ! Amount in rest of body (mg)
CR  = AR/VR                         ! Concentration in rest of body (mg/L)
CVR = CR/PR                         ! Rest of body venous blood concentration (mg/L0

! PLACENTA
RPla  = QPla*(CA*Free-CVPla*Free) &
       -rtrans1+rtrans2             ! Rate of amount in placenta (mg/h)
APla  = integ(RPla,0.0)             ! Amount in placenta (mg)
CPla  = APla/VPla                   ! Concentration in placenta (mg/L)
CVPla = CPla/PPla                   ! Placenta venous blood concentration (mg/L)

! PLASMA
RPlas    = (QFat*CVFat*Free) &
           +((QL+QG)*CVL*Free) &
           +(QR*CVR*Free) &
           +(QSk*CVSk*Free) &
           +(QK*CVK*Free) &
           +(QMam*CVMam*Free) &
           -(QC*CA*Free) &
           +(QPla*CVPla*Free) &
```

```
          +IVHOURLYDOSE &
          -Qfil*Ca*Free              ! Rate of amount free in plasma (mg/h)
APlas    = integ(RPlas,Aplas0)       ! Amount free in plasma (mg)
CAFree   = APlas/VPlas               ! Free concentration in plasma (mg/L)
CA       = CAFree/Free               ! Total concentration in plasma (mg/L)
APlastot = CA*VPlas                  ! Amount total in plasma (mg)
AUCPlas  = integ(CA,0.0)             ! Area under the curve of Ca vs. time (mg*h/L)

IV_TOTAL = integ(IVHOURLYDOSE,0.0)  ! Total IV dose to the mother (mg)

!-- FETUS --!

! Transfer to fetus
rtrans1 = ktrans1*CVPla*Free        ! Transfer from placenta to fetal plasma (mg/h)
Atrans1 = integ(rtrans1,0.0)        ! Amount transferred to fetal plasma (mg)
rtrans2 = ktrans2*Ca_F*FreeF        ! Transfer from fetal plasma to placenta (mg/h)
Atrans2 = integ(rtrans2,0.0)        ! Amount transferred to placenta (mg)

! REST OF FETAL BODY
RRF = QRF*(CA_f*FreeF-CVRF*FreeF) &
      -rtrans3+rtrans4              ! Rate of amount in fetal rest of body (mg/h)
ARRF = integ(RRF,0.0)               ! Amount in fetal rest of body (mg)
CRF = ARRF/(VRF+1.0e-33)            ! Concentration in fetal rest of body (mg/L)
CVRF = CRF/PRF                      ! Fetal rest of body venous blood concentration (mg/L)

! AMNIOTIC FLUID
RAmF = rtrans3-rtrans4              ! Rate of amount in amniotic fluid (mg/h)
AAmf = integ(RAmf, 0.0)             ! Amount in amniotic fluid (mg)
CAmf = AAmf/(Vaf+1.0e-7)            ! Concentration in amniotic fluid (mg/L)

rtrans3 = ktrans3*CVRF*FreeF        ! Transfer from fetal rest of body to amniotic fluid (mg/h)
Atrans3 = integ(rtrans3,0.0)        ! Amount transferred to amniotic fluid (mg)
rtrans4 = ktrans4*CAmF              ! Transfer from amniotic fluid to fetal rest of body (mg/h)
Atrans4 = integ(rtrans4,0.0)        ! Amount transferred to fetal rest of body (mg)

! FETAL PLASMA
RPlasF    =(QRF*CVRF*FreeF) &
          -(QRF*CA_f*FreeF) &
          +rtrans1-rtrans2          ! Rate of amount in fetal plasma (mg/h)
APlasF    = integ(RPlasF,0.0)       ! Amount free in fetal plasma (mg)
CA_f_free = APlasF/(VPlasF+1.0e-7)  ! Free concentration in fetal plasma (mg/L)
CA_f      = CA_f_free/FreeF         ! Total concentration in fetal plasma (mg/L)
APlastotF = CA_f*VPlasF             ! Amount total in fetal plasma (mg)
```

```
AUCPLASF  = integ(CA_f,0.0)         ! Area under the curve Ca_f vs. time (mg*h/L)

!---------------------------------------------------------------------------------------------------
! Dose balance check
BALANCE_DOSE = (IV_TOTAL+APLAS0+AG0+AL0+AFAT0+AK0+AR0+AMAM0+ASK0) &
               -(APLAS+AG+AL+AFAT+AK+AR+AMAM+ASK+APLA+APLASF+AFIL+ADELAY+AURINE+ARRF+AAMF)

!---------------------------------------------------------------------------------------------------
! Stop the simulation
schedule stopthesim .xn. (TSTOP-t)  ! When TSTOP-simulation time becomes negative ...
discrete stopthesim                 ! Discrete event stopthesim is "true"
TERMT(.true.)                       ! When stopthesim is "true", stop the simulation
end

END ! Derivative
END ! DYNAMIC
END ! PROGRAM
```

```
% MODEL AUTOMATION SCRIPT FOR ACSLX (AEGIS TECHNOLOGIES GROUP, INC, HUNSTVILLE, AL, USA)

%-----------------------------------------------------------------------------------------%
%  Monte Carlo script for the PFAS_GESTATION_MC.csl model code                            %
%  Coded by Marc-Andre Verner                                                             %
%-----------------------------------------------------------------------------------------%

output @clear
prepare @clear @all

%-----------------------------------------------------------------------------------------%
% Turning off verbose diagnostic output
WESITG=0;
WXDITG=0;
WEDITG=0;
CJVITG=0;
WNDITG=0;

%----------------------------------------------------------------------------------------
% Simulation parameters
TSTOP = 6570   ;
CINT  = 10000  ;
MINT  = 0.1    ;
MAXT  = 10000  ;
IALG  = 15     ;

sampled_values_MC_PFOS = [];
sampled_values_MC_PFOA = [];
VALUES_BIRTHWEIGHT_G   = [];

NUMITS = 250000;

seedrnd(123456789);

for x = 1 : NUMITS;

      disp(sprintf("Iteration #%d of %d", x, NUMITS));
      disp("----------------------------");

% Sensitive parameter distributions
PFOS_PL    = normrnd(3.72, 0.558, 2.604, 4.836)       ;
PFOA_PL    = normrnd(2.2, 0.33, 1.54, 2.86)           ;
PFOS_PR    = normrnd(0.2, 0.03, 0.14, 0.26)           ;
```

```
PFOA_PR    = normrnd(0.12, 0.018, 0.084, 0.156)       ;
PFOS_FREE  = normrnd(0.025, 0.00375, 0.01750, 0.03250);
PFOA_FREE  = normrnd(0.020, 0.00300, 0.00140, 0.02600);
PFOS_FREEF = normrnd(0.025, 0.00375, 0.01750, 0.03250);
PFOA_FREEF = normrnd(0.020, 0.00300, 0.00140, 0.02600);
PFOS_TMC   = normrnd(3.5, 0.525, 2.45, 4.55)          ;
PFOA_TMC   = normrnd(10.0, 1.5, 7.0, 13.0  )          ;
PFOS_KT    = normrnd(0.023, 0.00345, 0.0161, 0.0299)  ;
PFOA_KT    = normrnd(0.055, 0.00825, 0.0385, 0.0715)  ;
BWINIT     = normrnd(74.7, 17.8, 50.0, 114.0)         ;
VLC        = normrnd(0.026, 0.0039, 0.0182, 0.0338)   ;

% Calculation of birth weight (kg) based on GFR
RATIO_GFR     = normrnd(1.0, 0.246, 0.51, 1.49) ;
CONSTANT_REG  = 3.376 ;
BETA_REG      = 0.1755 ;
RESIDUAL      = normrnd(0.0, 0.441, -0.882, 0.882) ;
BIRTHWEIGHT   = BETA_REG*RATIO_GFR+CONSTANT_REG+RESIDUAL ;
BIRTHWEIGHT_g = BIRTHWEIGHT*1000 ;

%------------------------------------------------------------------------------------
% Simulations for PFOA
PFOS = 0 ;
PFOA = 1 ;

% Distibutions of blood levels in studies
AM    = 0.00253 ;
CV    = 0.446   ;
ASD   = AM*CV   ;
MIN   = 0.00001 ;
MAX   = 1.00000 ;

% Blood level parameters
CVINIT = normrnd(AM, ASD, MIN, MAX) ;

start @nocallback

% Record blood levels
initial_cv      = CVINIT;
cv_at_sample1   = CV_SAMPLE1 ;
cv_at_sample2   = CV_SAMPLE2 ;
cv_at_sample_m1 = CV_SAMPLE_M1 ;
cv_at_sample_m2 = CV_SAMPLE_M2 ;
```

```
cv_at_sample_m3 = CV_SAMPLE_M3 ;
cv_at_sample_m4 = CV_SAMPLE_M4 ;
cv_at_sample_m5 = CV_SAMPLE_M5 ;
cv_at_sample_m6 = CV_SAMPLE_M6 ;
cv_at_sample_m7 = CV_SAMPLE_M7 ;
cv_at_sample_m8 = CV_SAMPLE_M8 ;
cv_at_sample_m9 = CV_SAMPLE_M9 ;
cord_level      = CORD_SAMPLE ;
t_final         = T;

% Save PFOA results
if(abs(T-TSTOP)<1)
sampled_values_MC_PFOA = [sampled_values_MC_PFOA;  t_final cv_at_sample1 cv_at_sample2 cord_level ...
                          initial_cv PFOS_FREE PFOS_FREEF ...
                          PFOA_TMC PFOA_KT PFOA_K1C PFOA_K2C BWINIT RATIO_GFR RESIDUAL ...
                          cv_at_sample_m1 cv_at_sample_m2 cv_at_sample_m3 cv_at_sample_m4 ...
                          cv_at_sample_m5 cv_at_sample_m6 cv_at_sample_m7 cv_at_sample_m8 ...
                          cv_at_sample_m9 BIRTHWEIGHT_g];
end


%------------------------------------------------------------------------------------
% Simulations for PFOS
PFOS = 1 ;
PFOA = 0 ;

% Distibutions of blood levels in studies (from arithmetic means and stds)
AM    = 0.01302 ;
CV    = 0.368   ;
ASD   = AM*CV   ;
MIN   = 0.00001 ;
MAX   = 1.00000 ;

% Blood level parameters
CVINIT = normrnd(AM, ASD, MIN, MAX);

start @nocallback

% Record blood levels
initial_cv      = CVINIT;
cv_at_sample1   = CV_SAMPLE1 ;
cv_at_sample2   = CV_SAMPLE2 ;
cv_at_sample_m1 = CV_SAMPLE_M1 ;
```

```
cv_at_sample_m2 = CV_SAMPLE_M2 ;
cv_at_sample_m3 = CV_SAMPLE_M3 ;
cv_at_sample_m4 = CV_SAMPLE_M4 ;
cv_at_sample_m5 = CV_SAMPLE_M5 ;
cv_at_sample_m6 = CV_SAMPLE_M6 ;
cv_at_sample_m7 = CV_SAMPLE_M7 ;
cv_at_sample_m8 = CV_SAMPLE_M8 ;
cv_at_sample_m9 = CV_SAMPLE_M9 ;
cord_level      = CORD_SAMPLE ;
t_final         = T;

% Save PFOS results
if(abs(T-TSTOP)<1)
sampled_values_MC_PFOS = [sampled_values_MC_PFOS;  t_final cv_at_sample1 cv_at_sample2 cord_level ...
                          initial_cv PFOS_FREE PFOS_FREEF ...
                          PFOS_TMC PFOS_KT PFOS_K1C PFOS_K2C BWINIT RATIO_GFR RESIDUAL ...
                          cv_at_sample_m1 cv_at_sample_m2 cv_at_sample_m3 cv_at_sample_m4 ...
                          cv_at_sample_m5 cv_at_sample_m6 cv_at_sample_m7 cv_at_sample_m8 ...
                          cv_at_sample_m9 BIRTHWEIGHT_g];
end
end


save sampled_values_MC_PFOA @file=MC_PFOA_250K.csv @format=ascii @separator=comma
save sampled_values_MC_PFOS @file=MC_PFOS_250K.csv @format=ascii @separator=comma
```

References

Apelberg BJ, Witter FR, Herbstman JB, Calafat AM, Halden RU, Needham LL, et al. 2007. Cord serum concentrations of perfluorooctane sulfonate (PFOS) and perfluorooctanoate (PFOA) in relation to weight and size at birth. Environ Health Perspect 115(11): 1670-1676.

Chen MH, Ha EH, Wen TW, Su YN, Lien GW, Chen CY, et al. 2012. Perfluorinated compounds in umbilical cord blood and adverse birth outcomes. PLoS One 7(8): e42474.

Fei C, McLaughlin JK, Tarone RE, Olsen J. 2007. Perfluorinated chemicals and fetal growth: a study within the Danish National Birth Cohort. Environ Health Perspect 115(11): 1677-1682.

Halldorsson TI, Rytter D, Haug LS, Bech BH, Danielsen I, Becher G, Henriksen TB, Olsen SF. 2012. Prenatal exposure to perfluorooctanoate and risk of overweight at 20 years of age: a prospective cohort study. Environ Health Perspect 120: 668-673.

Hamm MP, Cherry NM, Chan E, Martin JW, Burstyn I. 2010. Maternal exposure to perfluorinated acids and fetal growth. J Expo Sci Environ Epidemiol 20(7): 589-597.

Inoue K, Okada F, Ito R, Kato S, Sasaki S, Nakajima S, Uno A, Saijo Y, Sata F, Yoshimura Y, Kishi R, Nakazawa H. 2004. Perfluorooctane sulfonate (PFOS) and related perfluorinated compounds in human maternal and cord blood samples: assessment of PFOS exposure in a susceptible population during pregnancy. Environ Health Perspect 112: 1204-1207.

Kim S, Choi K, Ji K, Seo J, Kho Y, Park J, Kim S, Park S, Hwang I, Jeon J, Yang H, Giesy JP. 2011. Trans-placental transfer of thirteen perfluorinated compounds and relations with fetal thyroid hormones. Environ Sci Technol 45: 7465-7472.

Maisonet M, Terrell ML, McGeehin MA, Christensen KY, Holmes A, Calafat AM, et al. 2012. Maternal concentrations of polyfluoroalkyl compounds during pregnancy and fetal and postnatal growth in British girls. Environ Health Perspect 120(10): 1432-1437.

Monroy R, Morrison K, Teo K, Atkinson S, Kubwabo C, Stewart B, Foster WG. 2008. Serum levels of perfluoroalkyl compounds in human maternal and umbilical cord blood samples.
Environ Res 108: 56-62.

Nolan LA, Nolan JM, Shofer FS, Rodway NV, Emmett EA. 2009. The relationship between birth weight, gestational age and perfluorooctanoic acid (PFOA)-contaminated public drinking water. Reprod Toxicol 27: 231-238.

Savitz DA, Stein CR, Bartell SM, Elston B, Gong J, Shin HM, Wellenius GA. 2012a. Perfluorooctanoic acid exposure and pregnancy outcome in a highly exposed community. Epidemiology 23: 386-392.

Savitz DA, Stein CR, Elston B, Wellenius GA, Bartell SM, Shin HM, Vieira VM, Fletcher T. 2012b. Relationship of perfluorooctanoic acid exposure to pregnancy outcome based on birth records in the mid-Ohio Valley. Environ Health Perspect 120: 1201-1207.

Stein CR, Savitz DA, Dougan M. 2009. Serum levels of perfluorooctanoic acid and perfluorooctane sulfonate and pregnancy outcome. Am J Epidemiol 170: 837-846.

Washino N, Saijo Y, Sasaki S, Kato S, Ban S, Konishi K, et al. 2009. Correlations between prenatal exposure to perfluorinated chemicals and reduced fetal growth. Environ Health Perspect 117(4): 660-667.

Whitworth KW, Haug LS, Baird DD, Becher G, Hoppin JA, Skjaerven R, et al. 2012. Perfluorinated compounds in relation to birth weight in the Norwegian Mother and Child Cohort Study. Am J Epidemiol 175(12): 1209-1216.
